# Massive Air Embolism in the Pulmonary Artery During Permanent Pacemaker Insertion

**DOI:** 10.7759/cureus.97639

**Published:** 2025-11-24

**Authors:** Neha Chopra, Kapil Mehta, Anwar Ansari, Saurabh Kumar Singh, Devesh Kumar

**Affiliations:** 1 Cardiology, Vardhman Mahavir Medical College and Safdarjung Hospital, New Delhi, IND; 2 Medicine, Atal Bihari Vajpayee Institute of Medical Sciences and Dr. RML Hospital, New Delhi, IND

**Keywords:** air embolism, durant manouver, hemodynamic collapse, pacemaker insertion, pulmonary artery

## Abstract

An elderly lady presented with syncope and was found to have complete heart block. She was planned for permanent pacemaker implantation the following day, and during the procedure, she developed an acute onset of dyspnea with hypotension, tachycardia, and her oxygen saturation on room air had dropped to 76%. After ruling out pneumothorax and myocardial infarction, we noticed that there was a massive air column floating in the pulmonary artery, which was visualized on cine-fluoroscopy. A provisional diagnosis of air embolism in the pulmonary artery was made, and she was managed with high-flow O2 and massive fluid boluses. Within 10 minutes, the patient improved with the disappearance of the air shadow on cine-fluoroscopy. Subsequently, the patient was discharged and improved dramatically.

## Introduction

Cardiac implantable electronic devices (CIEDs), including permanent pacemakers (PMs), cardiac resynchronization therapy devices with defibrillators (CRT-Ds) or without (CRT-Ps), and implantable cardioverter defibrillators (ICDs), are implanted worldwide in increasing numbers [1]. Complications after CIED treatment are associated with increased patient morbidity, healthcare costs, and increased mortality [2]. CIED complications are more frequent than generally acknowledged. Both patient- and procedure-related predictors may identify patients with a particularly high risk of complications. Most studies report risks of 5-6% for any complication and 3-4% for major complications after PM implantations [3]. In operators with a low annual volume(<50/year), complications due to CIED are much higher [4].

Massive air embolism is a rare, yet potentially fatal complication encountered during PM insertion. A better understanding of the pathophysiology would go a long way in avoiding this complication and managing it when inevitable. We discuss a rare case of air embolism in the pulmonary artery during pacemaker insertion and review the literature pertaining to it.

## Case presentation

**Figure 1:**
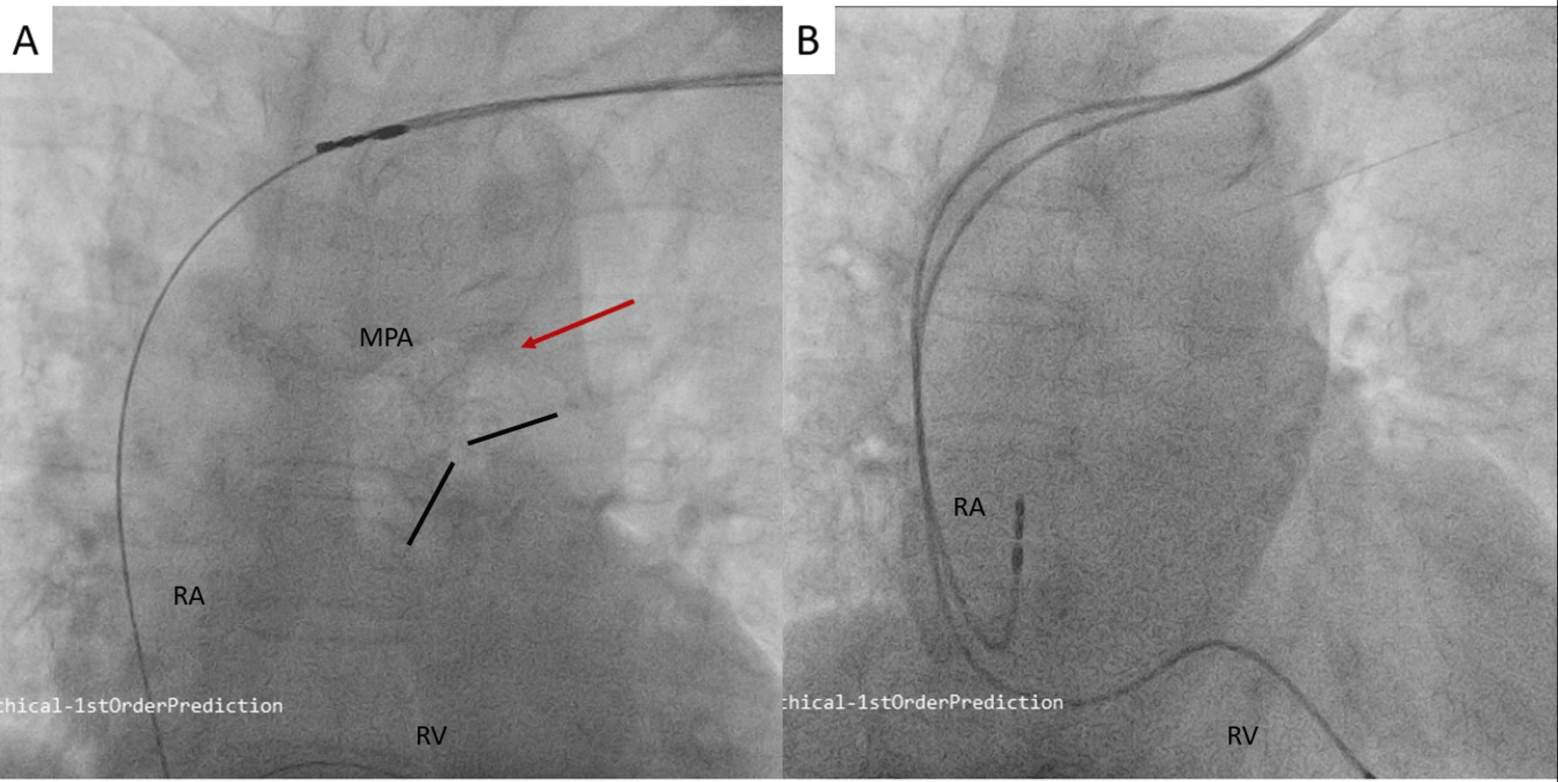
(A) The cine-fluoroscopy in the postero-anterior view with (red arrows) the air embolus in the main pulmonary artery. (B) The cine-fluorosocpy 10 minutes later after having given intravenous fluids and 100% oxygen to the patient showing disappearance of the air embolus from the main pulmonary artery.

An elderly lady in her 70s presented with recurrent episodes of syncope for one week. Twelve-lead electrocardiogram revealed complete heart block with a wide QRS (heart rate 36/minute), for which a transvenous temporary pacemaker was inserted through the right femoral vein, and she was planned for PM implantation the following day. She was hemodynamically stable, albeit extremely anxious, requiring administration of a low-dose opioid prior to the procedure (fentanyl 25 μg intravenously). After ensuring adequate anesthesia at the local site, a left-sided pre-pectoral pocket was prepared. Subsequently, the patient had begun snoring, though arousable on vocal stimulus. Two accesses through the left axillary vein were taken under fluoroscopic guidance. Once the dilator and guidewire complex was removed and exchanged with the pacing lead, she developed sudden dyspnea with hypotension (80/50 mmHg), tachycardia (120 beats/minute), and her oxygen saturation on room air had dropped to 76%. Pneumothorax, being the primary suspicion, was ruled out immediately on cine-fluoroscopy. However, on closer inspection of the fluoroscopy image, a large air embolus was noted in the proximal part of the main pulmonary artery (MPA) just above the pulmonary valve, leading to delineation of the MPA on fluoroscopy (Figure 1A and Video 1).

**Video 1:** Cine-fluoroscopy in antero-posterior (AP) projection showing a large air embolus in the main pulmonary artery just above the pulmonary valve.

This was determined to be a massive air embolism during the exchange of the dilator-guidewire complex with the pacemaker lead, despite the operator pinching the neck of the sheath (non-valved sheath). We attributed this to the deep inspiratory efforts of the patient and failure to hold her breath on command. She was immediately started on high-flow oxygen, and saline boluses were administered intravenously. She stabilized within minutes of treatment, and repeat fluoroscopy showed that the embolus had successfully dislodged from the origin of the MPA. The procedure was completed uneventfully thereafter, and she was subsequently discharged in a stable condition (Figure 1B).

## Discussion

Pacemaker implantation and its inherent complications are inevitable in the current era, given the use of sophisticated and bulkier devices [1]. While the number of implantations continues to increase, it comes at the cost of increased complications both early and late after CIED implantation [2]. Overall, the complication rate following CIED implantation is 5-6% and 3-4% of the patients encounter major complications [3,4]. These complications are classified into access-related complications, lead-related complications, and generator-related complications [5]. Amongst the access-related complications, pneumothorax, hemothorax, injury to the brachial plexus, and venous air embolism are the forerunners with respect to urgency and clinical significance [6]. 

Massive venous air embolism is a rare but potentially fatal complication during PM implantation. Intrathoracic pressure variation in central veins in different phases of respiration causes a sucking effect during inspiration, which may result in accidental air embolism. This risk is higher in patients afflicted with chronic obstructive pulmonary disease/interstitial lung disease, frequent cough, patients undergoing deep sedation, patients with large inspiratory efforts (due to large changes in intrathoracic venous pressure), dehydration, and inappropriate operator technique [7].

The clinical manifestation of air embolism has a myriad of presentations varying from asymptomatic to acute hemodynamic collapse, depending on the volume of air that embolizes. Historically, embolism of 300 mL or more of air has been known to be equivocally fatal [8]. It should be suspected in all patients who develop sudden-onset dyspnea or hemodynamic instability during the procedure, and in most cases is easily diagnosed on cine-fluoroscopy. It is prudent to remember that a patient with a left-to-right shunt, such as an atrial septal defect, is at risk of a cerebral embolism in case of such venous air embolisms [9]. 

Management involves prompt recognition and rapid resuscitation with intravenous fluids, 100% oxygen administration, and positioning in the Trendelenburg or left lateral position (Durant’s maneuver). 100% oxygen use is believed to create a pressure gradient for nitrogen escape, thus shrinking the offending bubbles [10]. Venous air embolism can lead to an “air trap” that tends to rise to the ventral - most structures in a supine patient - the right ventricular outflow tract (RVOT) and pulmonary artery due to the principle of buoyancy of air. Durant’s maneuver has been shown to improve survival in animal studies, likely by displacing the air trap to a larger volume chamber- the body and apex of the right ventricle, which is superior relative to the RVOT in this position [11]. If these simple maneuvers fail to dissipate the air, as a last resort, the pacing lead can be put across the pulmonary valve to disengage the air bubbles so as to create free passage for the forward flow of blood, which would help treat the right ventricular outflow obstruction caused by the bubbles. 

Venous air embolism is a largely preventable complication in expert hands. This case demonstrates the importance of swiftly exchanging the dilator-guidewire complex with the lead (while pinching the sheath) when inserting the lead. It is imperative that the patient is awake and asked not to take deep breaths during this crucial step. Lastly, a valved venous sheath should be used in high-risk cases [10,11]. It is critical to be aware of this complication and recognize it promptly, so that appropriate treatment may be instituted in a timely manner.

## Conclusions

Venous air embolism is a rare yet potentially fatal complication during CIED implantation. It should be suspected in patients with sudden-onset dyspnea during the procedure, especially once pneumothorax has been thoroughly excluded. It is an avoidable complication that can be avoided with utmost care and swiftness while exchanging the guidewire-dilator complex with the pacing lead, asking the patient to hold his breath while inserting the lead through the sheath, and by pinching the sheath at its neck.
